# Polypropylene Mesh Repair of Traumatic Hernia of the Vastus Lateralis: Case Report and Review

**DOI:** 10.1097/GOX.0000000000002101

**Published:** 2019-02-25

**Authors:** Patrick Meredith, Wenceslao M. Calonge

**Affiliations:** From the CIC (Centre d'Image Corporelle), Nyon, Switzerland.

## Abstract

Myofascial herniations of the lower limb are a rare cause of chronic nerve compression and pain. They may have congenital or traumatic origin, and the tibialis anterior muscle is the most frequent localization. A few cases will require operative management. An unusual case of symptomatic, acquired hernia of the vastus lateralis muscle in a young male basketball player is reported. After drainage of a compressive hematoma, the patient developed chronic pain and myositis of the vastus lateralis by friction against the edge of tensor fascia lata muscle. Secondary surgical reconstruction involved a polypropylene mesh repair 4 years after the initial trauma. This procedure has been described in a very small number of patients after iatrogenic lesions in total hip arthroplasty and on anterolateral thigh perforator flap donor site. Instead of denial and stoicism, this simple intervention could be proposed to patients as a therapeutic option.

## CASE REPORT

An 18-year-old basketball player suffered an impact on his left thigh during a Canadian university match on January 2011. The next day, he underwent a surgical decompression of a compartment syndrome of the left quadriceps. This included a lateral incision and a fasciotomy. The initial report of the surgeon at the time states that, due to important edema of the subdermal tissues, the skin wound was left open for 2 days and a staged closure took place on the second and fourth days after injury. He was discharged on the sixth day and resumed sports only 6 weeks after.

**Fig. 1.**
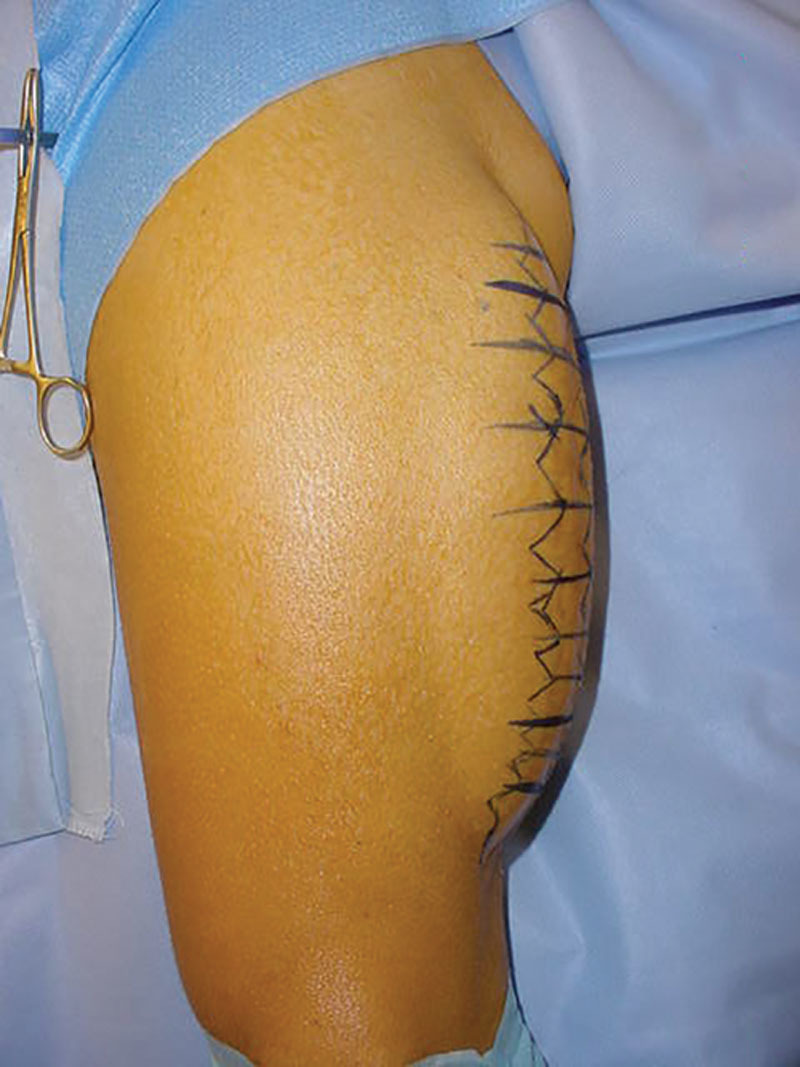
Preoperative marking of the left thigh.

He sought advice at our practice 7 months after the initial traumatism with local complaints of itching and redness and a persistent hump that was more noticeable with effort (Fig. 1). Initial therapeutic attitude consisted of neoprene compressive garments and small draining massages after performing sports. However, 4½ years after injury, he had stopped basketball practice and a painful edema had established.

**Fig. 2.**
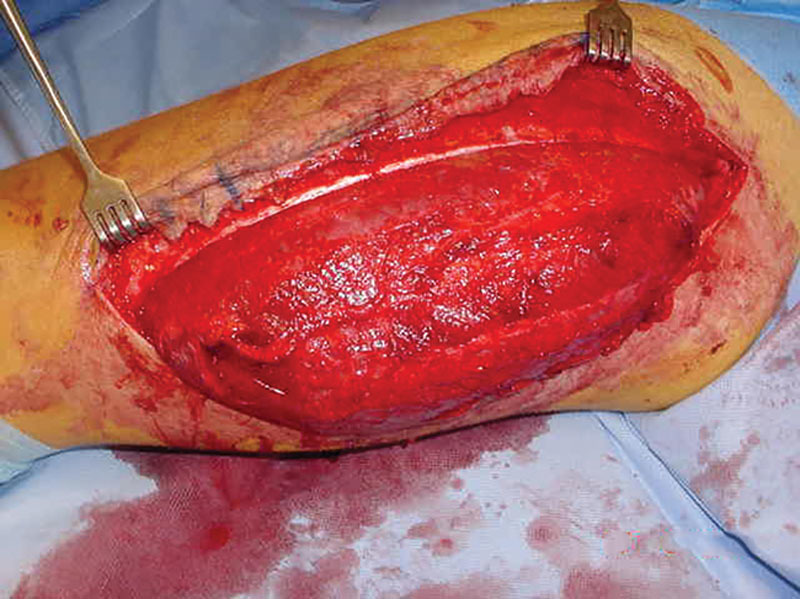
A maximum gap of 10 cm between the free edges of the injured fascia lata.

**Fig. 3.**
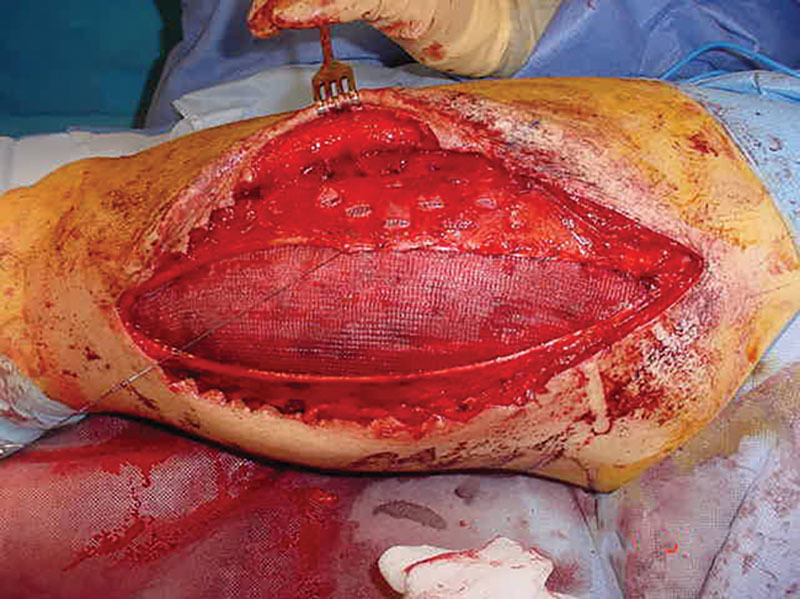
Multiple nonabsorbable sutures anchor a polypropylene mesh sheet bridging the gap. Small stab fenestrations on the remaining fascia lata allowed further stretching up to 3 cm on each side.

**Fig. 4.**
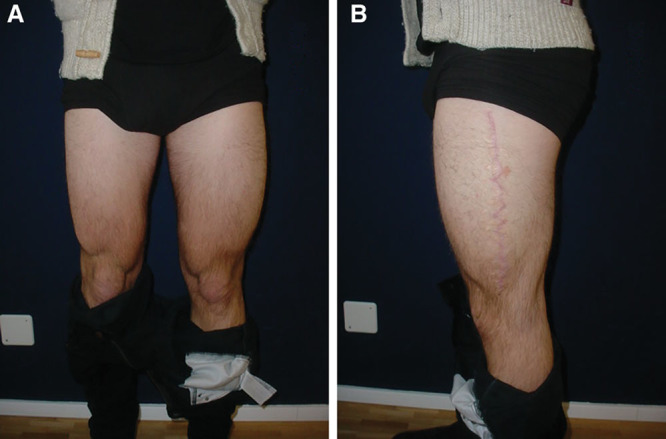
Frontal (A) and lateral (B) postoperative views of the patient.

A surgical revision of the scar and herniorrhaphy were proposed. Retraction and fibrosis of the open edges of fascia lata were clearly visible when detaching the skin. The gap measured about 10 cm (Fig. 2). A polypropylene mesh, the available material at our institution for large abdominal hernia repairs, was anchored by means of multiple, nonabsorbable stitches (Fig. 3). Some small fenestrations were incised on the “healthy” fascia lata to induce further punctual fibrosis and adhesions. The initial scar was excised and the skin was closed by several Y-V plasties. The patient was advised to wear a compressive garment for 6 weeks. He resumed light physical activity after 3 months. Pain and edema were absent at 5-month intermediary control, so he resumed sports 6 months after operation (Fig. 4). He had regained full competition at the time of a routine follow-up at 2 years.

## DISCUSSION

The repair of inguinal hernias with prosthetic meshes dates back to 1959, but reports about hernias of the lower extremity are rare until the 1990s.^1,2^

Surprisingly, references in literature for the specific localization of hernias of the vastus lateralis muscle through an injured fascia lata are even scarcer. Higgs et al.^3^ in 1995 reported a series of 6 patients (out of a series of 780 patients) who presented with a symptomatic fascia lata defect within the first 5 months following hip arthroplasty. The patients could perfectly localize a painful point that appeared during motion and were relieved by thigh stockings. The defect was clearly palpable by the examiners.

Richards et al.^4^ in 1998 described 2 patients who exemplify typical clinical situations: a 63-year-old man after a hip replacement and a 28-year-old rugby player who had suffered a high impact tackle.

First described in 1984, the anterolateral thigh flap epitomises another potential etiology. Damage to the fascia lata and vastus lateralis muscles was thoroughly studied as potential complications of the anterolateral thigh flap in a series of 37 patients by Kimata et al.^5^ in 2000. Lipa et al.^6^ in 2005 mention 6 cases of muscle herniation in a series of 21 consecutive anterolateral thigh flaps. In a large series of 220 patients, Hanasono et al.^7^ refer only 5 cases (2%) of wound dehiscence without any differentiation of the level of dehiscence. There is no mention of the term “hernia,” but the authors advocate the use of skin grafting to avoid wound dehiscence and compartment syndrome. Similarly, a recent comparison between medial (n = 155) and lateral (n = 197) thigh flaps^8^ does not differentiate the level of the dehiscence (11.2%) of the 197 lateral thigh flaps. Odili et al.^9^ would seem to be the first to report a single case of mesh repair of this insidious complication in 2009.

Given the thousands of traumatic events and hip arthroplasties and anterolateral thigh perforator flaps that are carried on every year, we assume that this condition goes mostly unreported. Ultrasonography could be an adjuvant diagnostic technique in case of doubt.^10^ Benefits and risks of a repair should be weighed for each individual case, but handicap was clear in our patient.
